# A genome-wide integrative study of microRNAs in human liver

**DOI:** 10.1186/1471-2164-14-395

**Published:** 2013-06-13

**Authors:** Eric R Gamazon, Federico Innocenti, Rongrong Wei, Libo Wang, Min Zhang, Snezana Mirkov, Jacqueline Ramírez, R Stephanie Huang, Nancy J Cox, Mark J Ratain, Wanqing Liu

**Affiliations:** 1 Department of Medicine, The University of Chicago, Chicago IL60637, USA; 2 Current affiliation: Institute for Pharmacogenomics & Individualized Therapy, University of North Carolina, Chapel Hill, NC 27599, USA; 3 Department of Medicinal Chemistry and Molecular Pharmacology, College of Pharmacy, Purdue University, West Lafayette, IN 47907, USA; 4 Department of Statistics, Purdue University, West Lafayette, IN 47907, USA

**Keywords:** microRNA, eQTL, Transcriptome, Genome-wide, SNP, Human liver, Pharmacogenetics

## Abstract

**Background:**

Recent studies have illuminated the diversity of roles for microRNAs in cellular, developmental, and pathophysiological processes. The study of microRNAs in human liver tissue promises to clarify the therapeutic and diagnostic value of this important regulatory mechanism of gene expression.

**Results:**

We conducted genome-wide profiling of microRNA expression in liver and performed an integrative analysis with previously collected genotype and transcriptome data. We report here that the Very Important Pharmacogenes (VIP Genes), comprising of genes of particular relevance for pharmacogenomics, are under substantial microRNA regulatory effect in the liver. We set out to elucidate the genetic basis of microRNA expression variation in liver and mapped microRNA expression to genomic loci as microRNA expression quantitative trait loci (miR-eQTLs). We identified common variants that attain genome-wide significant association (p < 10^-10^) with microRNA expression. We also found that the miR-eQTLs are significantly more likely to predict mRNA levels at a range of p-value thresholds than a random set of allele frequency matched SNPs, showing the functional effect of these loci on the transcriptome. Finally, we show that a large number of miR-eQTLs overlap with SNPs reproducibly associated with complex traits from the NHGRI repository of published genome-wide association studies as well as variants from a comprehensive catalog of manually curated pharmacogenetic associations.

**Conclusion:**

Our study provides important insights into the genomic architecture of gene regulation in a vital human organ, with important implications for our understanding of disease pathogenesis, therapeutic outcome, and other complex human phenotypes.

## Background

Gene expression variation has been shown to be important for the etiologies of common disorders including cancers [1], neuropsychiatric diseases [2], and various autoimmune disorders [3]. Thus, the identification of genetic polymorphisms, in the form of expression quantitative trait loci (eQTLs) [4], that have a functional impact on the regulation of gene expression provides a powerful means to characterize the molecular events responsible for disease pathogenesis and to inform potential therapeutic applications.

MicroRNAs (miRNAs), a class of conserved non-coding RNA molecules produced by a multi-step biogenesis pathway, have been shown to be a fundamental mechanism of gene expression regulation, targeting the 3′ untranslated region (UTR) of specific target messenger RNAs (mRNAs) for endonucleolytic cleavage or translational repression. In contrast to mRNAs, miRNAs are processed into duplexes by nuclear and cytosolic RNase III enzymes (Drosha and Dicer) in a maturation process. First identified in *Caenorhabditis elegans*[5], miRNAs have been implicated in key aspects of cellular, developmental, and pathophysiological processes. Studies have illuminated the roles of miRNAs in diverse biological phenomena, including cell proliferation and apoptosis [6], developmental timing of stage-specific cell lineages [7], the patterning of tissues in the developing embryo [8], and the regulation of immune response to pathogens [9].

The functional characterization of miRNAs is currently an active area of investigation. In this study, we sought to contribute to the functional understanding of miRNAs by performing genome-wide expression profiling in human liver. The liver is the primary organ in xenobiotic disposition, through a complex system involving a variety of drug transporters and metabolizing enzymes. Thus, patterns of gene expression in liver are likely to influence the systemic availability of xenobiotics, mediating downstream pharmacologic effects. Furthermore, studies of the liver transcriptome are likely to reveal important insights into liver physiology and disease processes. Recent studies have shown that miRNAs are abundant in the liver and regulate a broad spectrum of liver functions [10]. These biomolecules may serve as diagnostic markers for such liver diseases as hepatocellular cancer [11] and polycystic liver diseases [12], or as promising therapeutic targets (for example, for the chemically engineered oligonucleotides, called “antagomirs”, designed to be specific silencers of endogenous miRNAs *in vivo*) [13].

Thus, we conducted a genome-wide integrative study of miRNAs in human liver with the purpose of clarifying their functional impact on the transcriptome and on complex human traits. We identified a comprehensive list of miRNAs abundantly expressed in liver. We sought to dissect the genetic basis of miRNA expression variation in a tissue of direct relevance to many human diseases and pharmacologic phenotypes. We applied quantitative trait loci (QTL) mapping to characterize genetic regulation of miRNA expression levels as quantitative traits. Given the relevance of the tissue for drug metabolism, we identified miRNAs significantly correlated with the expression of the so-called Very Important Pharmacogenes (VIP Genes) as maintained by PharmGKB [14], comprising a list of genes of particular importance for drug response. Finally, this study provides biologic insights into certain findings from genome-wide association studies by establishing potential mechanistic links into replicated associations with a broad spectrum of complex traits.

## Results

### miRNA expression profiling

Genome-wide expression profiling (see Methods) identified 277 expressed miRNAs in liver, defined here as having non-missing expression values for at least 75% of the samples. Of these, 166 miRNAs had zero missing values. A missing value for a given sample may result when the calling of the particular miRNA failed. This “failure” meant that 2 or more of the 4 replicated measures of the miRNA were flagged 1 or 2 by the (Exiqon) image software, indicating that the quantified signal was below background. Alternatively, a missing value may result when the Hy3 and Hy5 signals were lower than 1.5 times that of the median signal intensity of the given slide.

The 166 miRNAs with no missing values include some of the most abundantly expressed miRNAs in liver, including miR-122, a liver-specific miRNA previously known to be expressed in liver tissue, human primary hepatocytes, and in cultured liver cells [15]. Included in these 166 miRNAs too are other miRNAs known to be abundantly expressed in adult liver tissue, including miR-16, miR-27b, miR-30d, miR-126, as well as the let-7 family of miRNAs [10]. Additional file 1: Figure S1 is a heatmap illustrating a two-way hierarchical clustering [16] of miRNAs and samples.

### miRNA expression levels negatively correlated with putative target mRNAs

**Figure 1:**
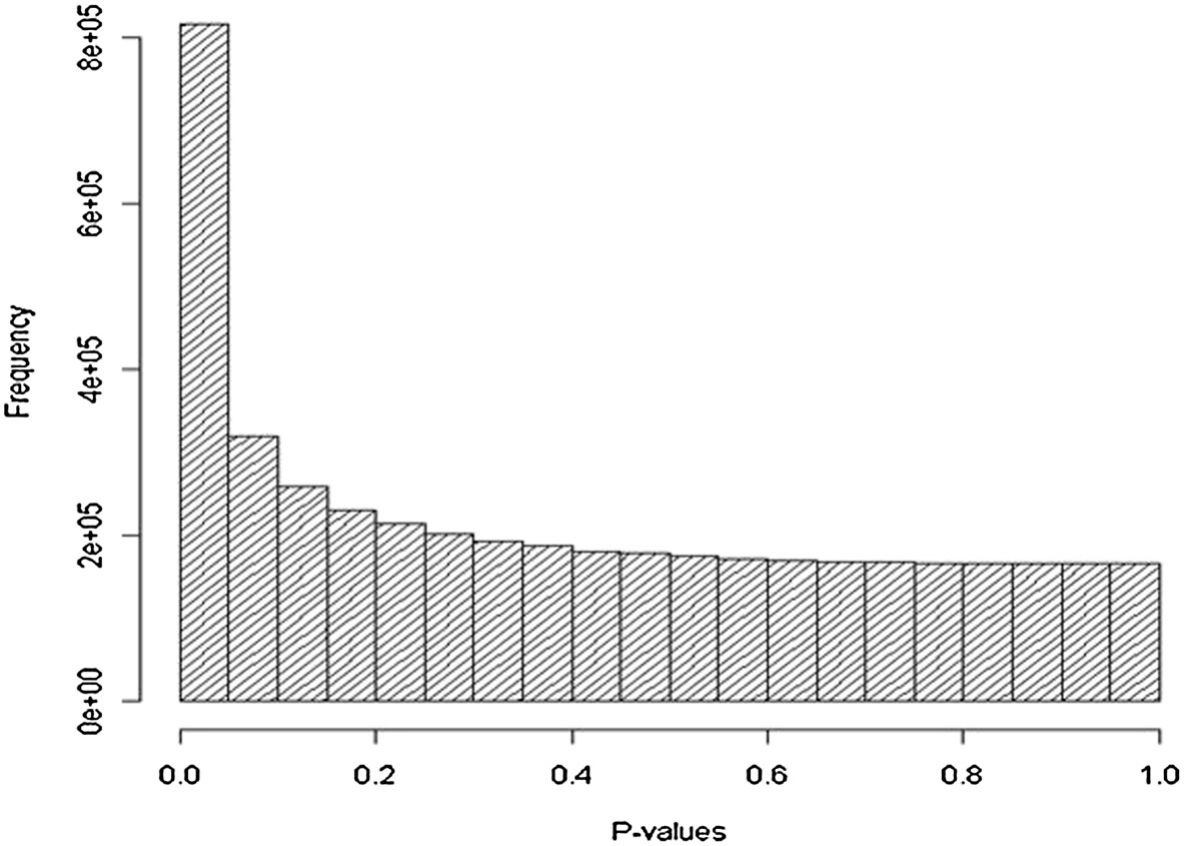
**Histogram of P-values for the negative associations between miRNA expression and mRNA expression.** Note the enrichment for low p-values, suggesting the presence of true signals.

Global baseline gene expression in liver on these samples was previously quantified using the Agilent 4x44 array [17] (see Methods for details). Figure 1 illustrates the distribution of p-values for the negative associations between miRNA expression and mRNA expression. (All correlation tests between miRNA expression and mRNA expression in our study involve the inverse relationship [i.e., negative beta], which is our primary interest here, unless explicitly stated.) The enrichment of low p-values among the miRNA-mRNA relationships suggests that our study is capturing some true signals and, of these, miRNAs tend to be associated with multiple mRNAs (as perhaps expected from the fact that miRNAs are known to target at least a third of all genes in the genome [18]). For multiple testing for the miRNA-mRNA (negative) correlations, we used a false discovery rate (FDR) approach [19]; we defined FDR < 0.05 as significant.

At this stringent threshold, we found 275 miRNAs (of the 277 expressed miRNAs) to be negatively correlated with mRNA expression (see Additional file 2: Table S1 for the list of the top miRNA-mRNA relationships); comparisons were made against 19,749 transcripts in this analysis. In particular, miR-122, is associated with 105 target mRNAs (all p < 10^-12^). Additional file 3: Table S2 lists the most significant of the miRNA-mRNA relationships and their overlap with computational miRNA target prediction approaches, miRBase [20] and ExprTarget [21].

Furthermore, we conducted functional enrichment analyses, using DAVID [22], on the genes that showed the most significant negative correlations with miRNAs in liver (p < 10^-10^) and found a highly significant excess (p = 0.02, Benjamini-Hochberg [23]) for genes (*N* = 25) involved in *cell adhesion*, characterized as the attachment of a cell, either to another cell or to an underlying substrate such as the extracellular matrix, via cell adhesion molecules [24].

### The genetic basis of miRNA expression in liver

**Figure 2:**
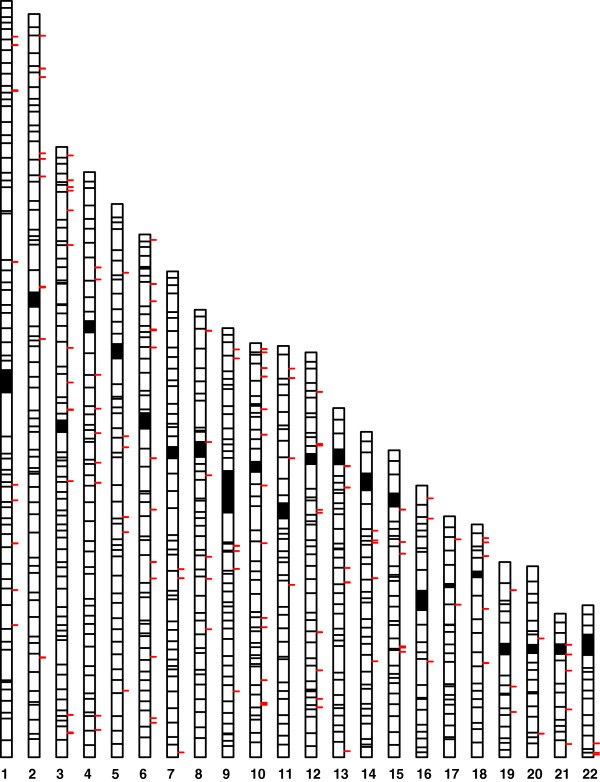
**A genome-wide map of miRNA eQTLs in liver.** Shown here are all SNPs with p<10^-6^ for association with miRNA expression.

**Figure 3:**
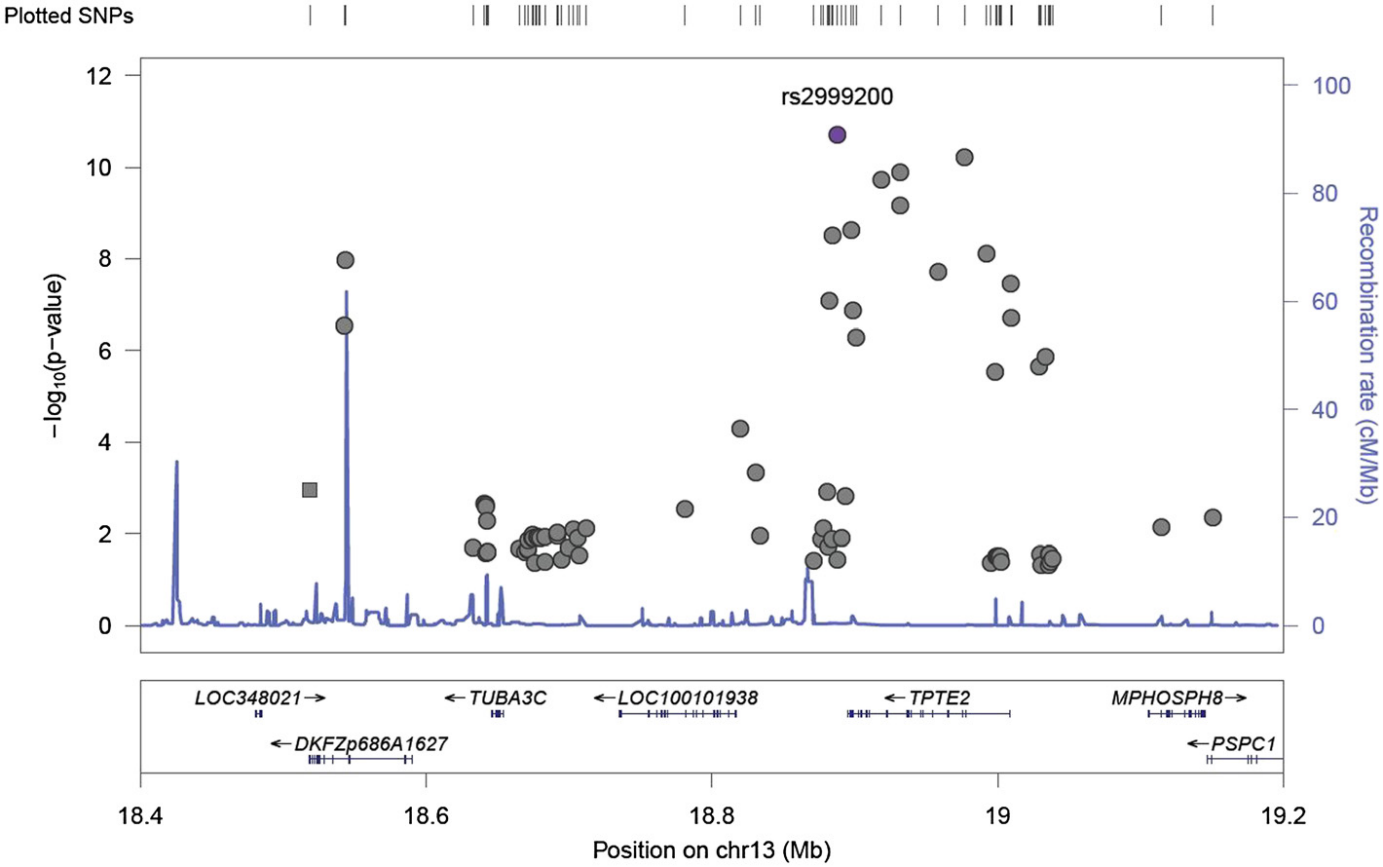
**A regional view of a genome-wide scan for miR-eQTLs.** Shown here is a regional plot that illustrates the eQTL mapping for miR-200c, a molecule that has been reported to successfully distinguish hepatocellular carcinoma from liver metastases.

We hypothesized that miRNA expression variation may in part be due to effects of genetic polymorphisms. We therefore conducted genome-wide association studies to map miRNA expression levels to genomic loci as miR-eQTLs. First we used the increased density of interrogated SNPs from conducting imputation with Bimbam [25] as previously described [17]. Using mean imputed genotypes for nearly 2 million SNPs and miRNA expression levels for the identified expressed genes in liver, we performed QTL mapping on each miRNA. Given our sample size, we considered only those SNPs that meet the minor allele frequency threshold of 15% in our QTL mapping. We used a strict Bonferroni threshold (based on the number of SNPs and the number of miRNAs tested, p < 10^-10^) to define a significant “trans” association. Despite this stringent threshold, we nevertheless found a genome-wide significant set of miR-eQTLs, including rs263418 for miR-938 (p = 4.1x10^-13^), rs2999200 for miR-200c (p = 1.9x10^-11^), and rs11088818 and miR-10b (p = 1.5x10^-11^). At a suggestive threshold (p < 10^-8^), we found 39 miRNAs (14% of all tested) to be associated with SNP genotypes (*N* = 18) (see Figure 2 for a genome-wide map of these miRNA-associated SNPs), including rs2999200 and rs6551952 for the abundantly expressed and liver-specific miR-122 (p = 7.7x10^-9^ and p = 3x10^-9^, respectively). Figure 3 provides, as an illustrative example, a regional view [26] of a genome-wide scan for miR-eQTLs for miR-200c, a molecule that has been reported to successfully distinguish hepatocellular carcinoma from liver metastases [27].

Several patterns emerge from this analysis. First, we identified some SNPs associated with multiple target miRNAs. For example, rs2999200 was found to be significantly associated with miR-198, miR-509-3-5p and miR-519e* (p = 8.8x10^-13^, p = 6.9x10^-11^ and p = 4.3x10^-13^, respectively). Second, we identified miRNAs associated with multiple SNPs (miR-10b and the SNPs rs11088818 at p = 1.5x10^-11^, rs11088887 at p = 1.7x10^-11^ and rs3778533 at p = 5.2x10^-9^), the last two of which are not in linkage disequilibrium.

### miR-eQTLs are enriched for mRNA eQTLs

We sought to further functionally characterize the miR-eQTLs obtained from our genome-wide mapping analysis. Our group had previously conducted whole-genome gene expression profiling in a larger set (*N* = 206) of these liver samples, which allowed us to determine to what extent the identified miR-eQTLs influence global gene expression as mRNA eQTLs [17]. In this previously reported eQTL study, 1,787 genes were found to have significant *cis*-linked genetic effects on expression levels, a large proportion of which were replicated in two other independent collections of human liver; furthermore, 353 gene expression traits were found to have significant *trans* eQTLs. We devised a simulation procedure to test for enrichment of mRNA eQTLs among miR-eQTLs. We asked whether SNPs associated with miRNA expression (minor allele frequency > 15%, p < 10^-6^) are enriched for SNPs associated with mRNA expression (defined as p < 10^-5^). Using 1000 randomly generated sets of SNPs (matching the minor allele frequency distribution of the miRNA-associated SNPs) as controls, we generated the empirical null distribution for the overlap count with the mRNA-associated SNPs. We observed that the miRNA-associated SNPs are more likely to be mRNA-associated (Additional file 4: Figure S2; enrichment p = 0.049) than a random set of allele frequency matched SNPs.

### miR- eQTLs and replicated associations from genome-wide association studies of disease susceptibility and quantitative traits

We hypothesized that the results of our miR-eQTL mapping might help to clarify many of the associations found in the NHGRI catalog of published genome-wide association analyses, most of which have been validated in a subsequent replication study. For the definition of miRNA-associated SNPs, we chose the liberal threshold p < 10^-4^, because we were interested in functionally annotating SNPs with information on miRNA expression and, furthermore, the SNPs had prior information on association with complex human phenotype. Additional file 5: Table S3 lists the overlap between miRNA eQTLs and GWAS SNPs; for every trait-associated SNP, it shows the target miRNAs, the p-value for the SNP-miRNA association, and the direction of effect. We found miRNA-associated SNPs for a broad spectrum of complex traits, including serum uric acid, QT-interval, pulmonary function, cognitive performance, weight and height, as well as a list of complex diseases such as Alzheimer’s disease, Crohn’s disease, ulcerative colitis, myocardial infarction, and multiple sclerosis. We found no excess of a particular direction of effect (plus or minus) for these trait- and miRNA- associated SNPs; that is, there is no tendency for the “risk allele” to be associated with lower or higher miRNA expression. Of note however, among these SNPs, we found several which were associated with the expression levels of multiple miRNAs (Additional file 5: Table S3), which were thus annotated to the same complex trait. This latter observation raised the hypothesis that trait- and miRNA- associated SNPs may indeed be more likely to regulate the expression levels of multiple miRNAs than allele frequency matched SNPs. Simulation analyses using 1000 randomly generated sets of SNPs (matching the minor allele frequency distribution of the trait- and miRNA- associated SNPs) in fact confirmed this to be the case (enrichment p = 0.01).

**Figure 4:**
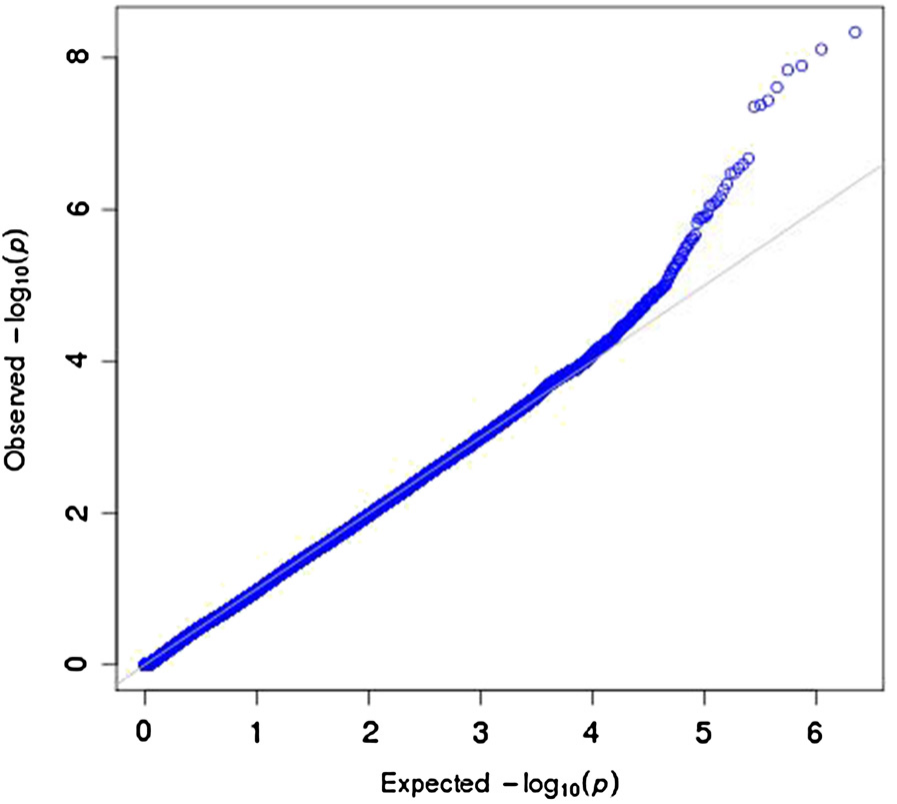
**QQ plot of associations between trait-associated SNPs and miRNA expression in liver.** We considered the associations between the trait-associated SNPs from the NHGRI catalog of genome-wide association studies and miRNA expression in liver and found an excess of significant regulatory signals on miRNA expression. Shown here is the global distribution of p-values from the association with miRNA expression for the NHGRI SNPs, with deviation from expectation for the most significant associations.

We asked whether the trait-associated SNPs in the NHGRI catalog are enriched for miRNA associations in liver. Figure 4 is a QQ plot that shows a significant excess of miRNA regulatory signals among the NHGRI catalog SNPs. The blue dots depict the distribution of miRNA association p-values for the trait-associated SNPs from the NHGRI catalog. The QQ plot includes all (tested) association p-values between trait-associated SNPs and miRNA expression (in particular, regardless of the directional effect of miRNA-mRNA pairings since this analysis is specifically concerned with identifying miRNA associations for the NHGRI catalog SNPs whether or not the miRNA regulates an mRNA and whether or not the miRNA is co-expressed with certain mRNAs in the tissue). Furthermore, the departure from expectation is observed for only the most significant SNP-miRNA pairs.

### VIP Genes and miRNA Regulation

**Figure 5:**
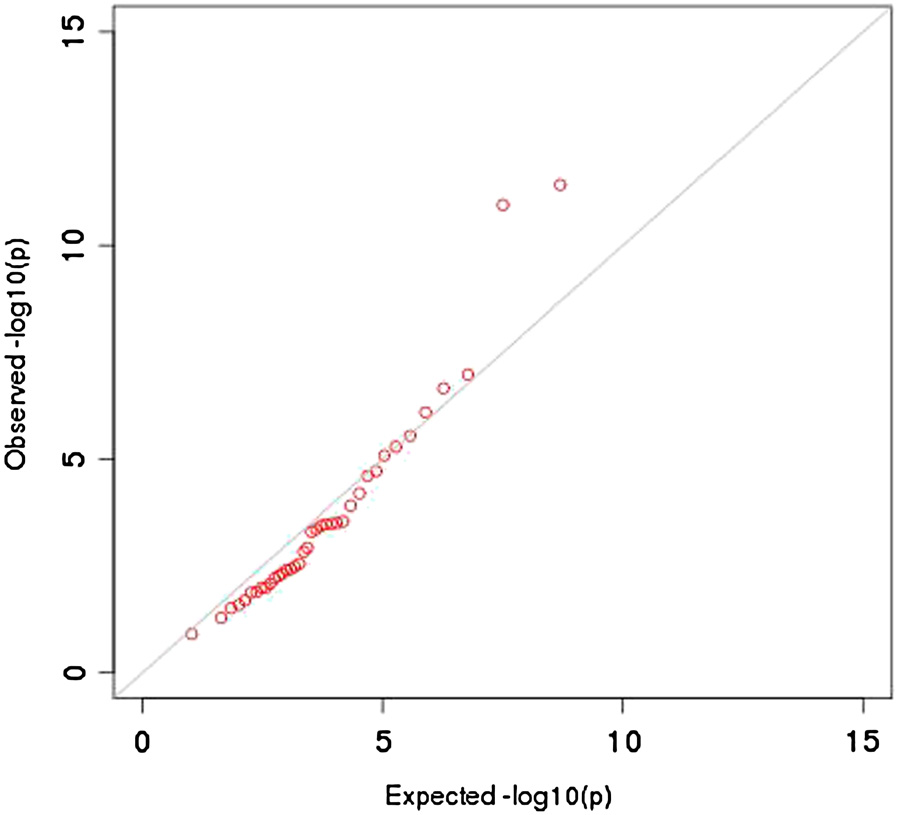
**VIP genes are under substantial miRNA regulation relative to the genomic background.** The QQ plot shows the association p-values between the VIP genes and miRNA expression traits for all (negatively correlated) miRNA-mRNA pairs. We compared the distribution of the best association p-value per gene for the VIP genes (“observed” data) to that of random sets (each of the same size as that of the VIP genes) of the most significant p-value per gene for the randomly selected genes (“expected” data). Only the negatively correlated miRNA-mRNA pairs were used in this analysis.

Given the aforementioned crucial role of liver in xenobiotic metabolism, we hypothesized that identifying genetic variations influencing miRNA expression, which in turn regulates the expression of the specific target mRNA(s) of the corresponding VIP gene(s), should highlight polymorphisms (and thereby associated genetic-based mechanisms) with potential functional impact, at the pharmacodynamic or pharmacokinetic level, on drug response. Pursuing this hypothesis, we first conducted a comprehensive evaluation of the association between miRNA expression and mRNA expression for each of the VIP genes. We conducted random sampling (n = 1,000) of the same size as that of the VIP genes and found, on the basis of comparisons of the median p-value, that no p-value distribution (from the negative correlations between the miRNAs and mRNAs) of any random set matches or exceeds that of the VIP genes (empirical p < 0.001). Figure 5 shows a QQ plot from the association p-values between miRNA expression and transcript (mRNA) level, for which increased (decreased) miRNA expression was associated with decreased (increased) mRNA expression. We compared the distribution of the best association p-value per gene for the VIP genes (“observed” data) to that of random sets of the most significant p-value per gene for the randomly selected genes (“expected” data). Again, in the “observed” and “expected” data, only the negative correlations between the miRNAs and mRNAs were used. Taken together, these results demonstrate that the VIP genes show substantial regulatory miRNA effect, indeed greater than expected by chance.

We found that 28 of the 41 VIP genes were significantly (and negatively) associated (FDR < 0.05) with miRNA expression in liver. Several miRNAs (for example miR-23b) were negatively associated (FDR < 0.05) with multiple VIP genes (e.g., *F5, ADRB1, GSTP1, KCNH2, KCNJ11, NQO1,* and *PTGIS*). Additional file 6: Table S4 shows the full list of these significant miRNA-VIP genes relationships. Notably, several of the relationships we identified (e.g., *ADRB1* and miR-30a, b, c, and e) were predicted by miRBase [20] as well as ExprTarget [21].

We investigated the miRNA effect (*β*) on the VIP genes relative to the global distribution of effect sizes for the negative associations (FDR < 0.05) between miRNAs and mRNAs (Additional file 7: Figure S3). This distribution showed a mean of *μ* = −1.21 and standard deviation of *ơ* = 1.05. We then considered those VIP genes with *β* < −4 to identify pharmacogenes showing substantial miRNA regulatory effect from the most significant of the miRNA-mRNA relationships (FDR < 0.05). We identified 12 (of 41) such VIP genes: *PTGIS*, *KCNH2*, *GSTP1*, *ADRB1*, *NQO1*, *ADRB2*, *F5*, *ABCB1*, *MTHFR*, *BRCA1*, *SLCO1B1*, and *DPYD*. This represents a significantly higher proportion relative to what is expected genome-wide (5%), demonstrating that these pharmacogenes are enriched for large miRNA regulatory effects.

### Clinical Associations in Pharmacogenetics and miR- eQTLs

Since the liver is the most important site of drug metabolism and excretion, we asked to what extent the identified miR-eQTLs may be used to clarify the mechanistic role of published genetic associations in a broad spectrum of pharmacologic traits. We thus compiled a list of such pharmacogenetic associations from PharmGKB (http://www.pharmgkb.org) [28] and from our own curation [29]. These clinical annotations are classified according to the strength of evidence for the association [28]. Level 1 requires replication in populations of at least 1,000 cases and 1,000 controls of the same ethnicity and corrected p-value < 0.05. Among these level-1 variants are the established associations rs12248560 (*CYP2C19*) for clopidogrel, rs1057910 (*CYP2C9*) and rs9923231 (*VKORC1*) for warfarin, and rs776746 (*CYP3A5*) for cyclosporine. Level 2 annotations require corrected p-value < 0.05 and at least one population of at least 100 although the variant may or may not be replicated. Among these are rs2284017 (*CACNG2*) for lithium (as treatment for Bipolar Disorder), rs1801252 (*ADRB1*) for atenolol (Coronary Artery Disease), and rs429358 (*APOC1*, *APOE*) for ritonavir (HIV, HIV infections, Hyperlipidemias). Level 3 falls short of level 2 criteria due to sample size or p-value, or because the evidence is based on *in vitro*/pharmacokinetic (PK) support only. Consistent with this evidence-based annotation, we incorporated published results from genome-wide association studies of a wide array of chemotherapeutic agents, as cataloged in a public resource PACdb [29] we created. In total, 480 SNPs from all three levels were included.

We found that these clinical associations are enriched (enrichment p < 0.05) for miRNA-associated SNPs (p < 0.001) relative to frequency-matched SNPs. For example, SNPs that show evidence for regulating the expression of miRNAs in liver include several replicated clinical associations with response to chemotherapeutic agents, including rs9332377 (cisplatin; miR-619) [30] and rs4880 (cyclophosphamide; miR-199a-5p, miR-376a, miR-450a, miR-590-5p) [31]. We hypothesized that the miRNAs associated with these pharmacogenetic variants have significantly higher regulatory effect on their target genes. Additional file 8: Figure S4 compares the distribution of effect sizes on target mRNAs for the miRNAs associated (p < 0.001) with the pharmacogenetic variants and the remaining expressed miRNAs, indicating the larger effect sizes (in absolute value) of the former (p = 0.042, *t*-test). The larger effect sizes (in magnitude) on the target genes for the miRNAs associated with these pharmacogenetic associations become more significant (p = 0.0086) when we restrict only to those variants (*N* = 192) with the highest level of evidence (level 1 and level 2).

### Experimental Confirmation of Gene Expression, miRNA-mRNA Correlation and miR-eQTLs

**Figure 6:**
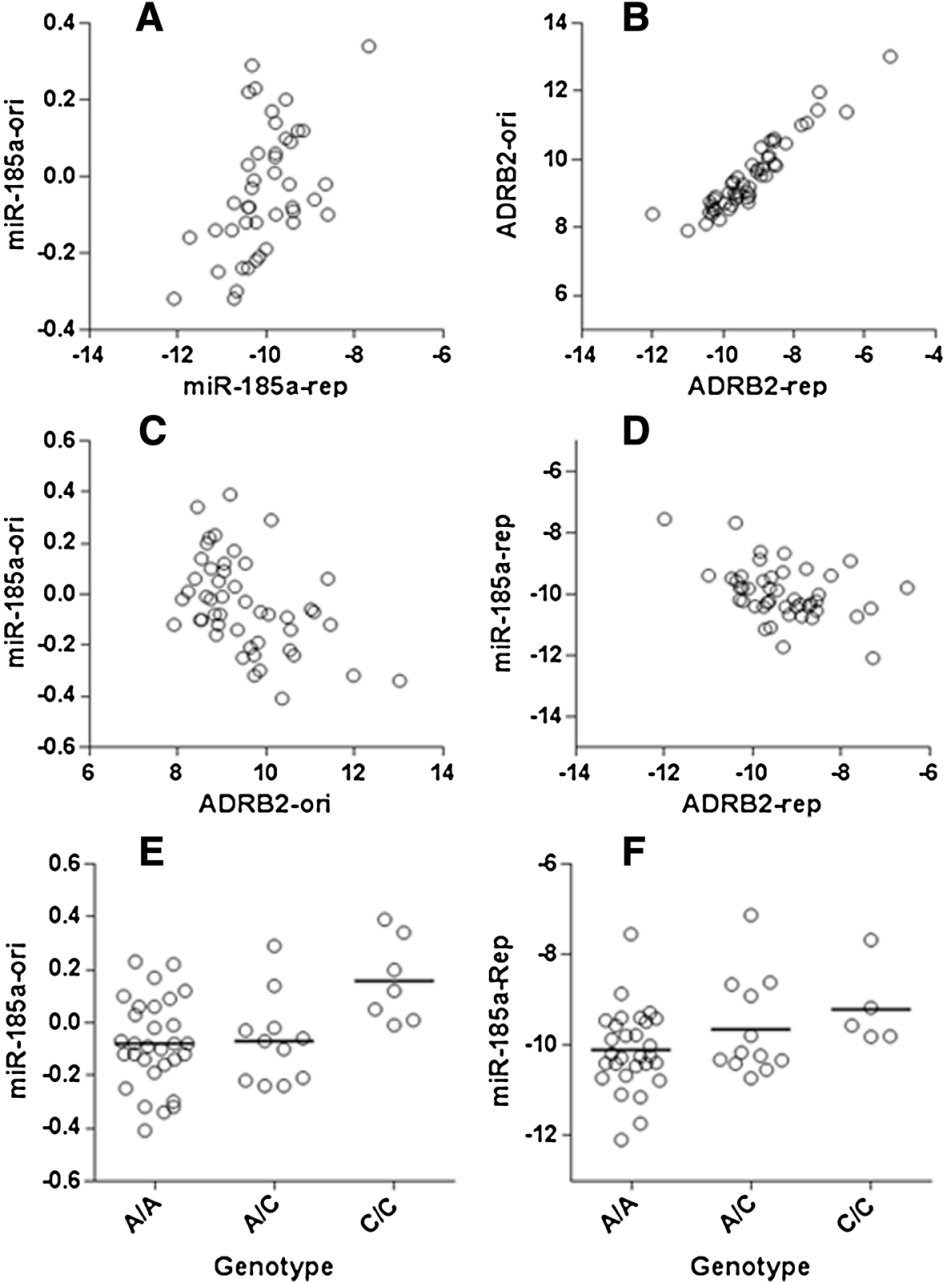
**Comparison of the miRNA expression (panel A), mRNA expression (panel B), and miRNA-mRNA correlations (panels C and D) between the replication (Q-PCR, labeled as “rep”) and the original (microarray, labeled as “ori”) datasets as well as confirmation of a microarray-identified miR-eQTL (panels E and F).** Data were plotted only for the samples with total RNA available (n = 53).

We chose two miRNAs (miR-148a and miR-185a), two mRNAs (*PTGIS* and *ADRB2*), and two miR-eQTLs (rs6551952 and rs1220) for additional experimental confirmation. In the aforementioned analysis, miR-148a and miR-185a were significantly inversely correlated with *PTGIS* (p = 1.14 × 10^-8^) and *ADRB2* (p = 5.02 × 10^-5^), respectively, while rs6551952 and rs1220 were significantly associated with miR-148a (p = 1.97 × 10^-7^) and miR-185a (p = 1.72 × 10^-7^), respectively. The two miRNAs and two mRNAs were quantified using Quantitative PCR (Q-PCR) in the samples for which RNA was still available (n = 53). We found that the correlations between the Q-PCR and microarray data in gene expression were generally high (r ≥ 0.48) (Table 1, Figure 6). In these 53 samples, both the correlation coefficient and the direction of the correlation between the miRNAs and the mRNAs were quite similar (Table 1). In confirming the two miR-eQTLs, there was only a limited number of samples in the heterozygote genotype class (n = 2) and the rare-allele homozygote genotype class (n = 2) at the rs6551952 locus. However, we did observe similar correlations between the genotype at rs1220 and the expression of miR-185a when comparing the Q-PCR and the microarray data (Table 1, Figure 6E and F).

**Table 1:** Confirmation of miRNA/mRNA expression, the miRNA-mRNA correlation and the miR-eQTLs

**Gene or SNP**		**Correlation (n = 53)**			
**Q-PCR**	**Microarray**	**Replication r**	**Replication p**	**Original r**	**Original p**
*miR-148a*	*miR-148a*	0.48	0.001	-	-
*miR-185a*	*miR-185a*	0.52	0.0003	-	-
*PTGIS*	*PTGIS*	0.5	0.0002	-	-
*ADRB2*	*ADRB2*	0.94	<1 × 10^-7^	-	-
*miR-148a vs PTGIS*		−0.36	0.016	−0.36	0.012
*miR-185a vs ADRB2*		−0.43	0.003	−0.46	0.001
*miR-185a vs rs1220*		0.32	0.029	0.39	0.006

The correlation coefficient (r) and statistical significance (p) of the comparisons between Q-PCR data and the original microarray data are shown. Analyses were based on the samples for which RNA was available (n = 53).

## Discussion

In this study, we performed large-scale integration of genomic information, transcriptome data, and miRNAome profiling in human liver. The resulting integrative map offers the possibility of identifying key regulatory pathways involved in disease biology and therapeutic outcome. Genome annotation of global mRNA and miRNA expression in this tissue should facilitate explorations of the complex interrelationships between genetic variation, the regulome consisting of the regulatory nodes and networks underlying biological function, and complex traits (pathophysiology and pharmacogenomic traits). The resource we have created expands on earlier studies of the heritability of miRNA expression in (transformed) lymphocytes [32] and of the utility of eQTL mapping in a variety of tissues for the identification of disease-associated genes [33,34].

This study presents a genome-wide analysis of miRNA expression in human liver, with a primary focus on understanding miRNA regulatory effects on the transcriptome, but also a special emphasis on obtaining pharmacogenomic insights from an exploration of gene regulation in *the* tissue of key importance for drug metabolism and excretion. Consistent with studies that show that miRNAs target a third of the genome [18], we found that 275 miRNAs are inversely correlated (FDR < 0.05) with the expression levels of 5,767 genes. This finding implies that mRNA expression in liver is likely to be altered by multiple miRNAs. In particular, we identified several miRNAs that significantly downregulate (FDR < 0.05) the so-called VIP genes, which are key pharmacogenes involved in modulating response to one or more drugs; these miRNAs are thus likely to have a significant pharmacodynamic or pharmacokinetic influence on drug response phenotypes. For example, miR-23b, which has been implicated in liver stem cell differentiation [35], significantly downregulates the expression of multiple VIP genes (FDR < 0.05), including *F5, ADRB1, GSTP1, KCNH2, KCNJ11, NQO1,* and *PTGIS.* In addition, we have shown that these important pharmacogenes are significantly enriched for large miRNA regulatory effects in liver, compared to genome-wide expectation.

We found, in a gene set enrichment analysis, that the genes with the most significant negative correlations with miRNAs in liver were enriched for cell adhesion molecules. Regulation of cell adhesion molecules has been shown to play a role in the pathogenesis of many human diseases [36] as well as in normal cellular and organismal homeostasis [37]. A growing body of literature (for review, see Commentary in [37]) has demonstrated the crucial role of miRNAs in four major adhesion processes: cytoskeletal dynamics, cell-cell adhesion, cell-matrix adhesion, and extracellular matrix; interestingly, as Robert Weinberg *et al.*[37] noted, those miRNAs that target genes belonging to more than one of these adhesion processes are notably the same miRNAs that have been implicated in various human diseases [38-40].

Our study demonstrates that miRNA expression in human liver has a significant genetic component. Most investigations of the effect of genetic variation on miRNAs have so far been focused on genetic influence (e.g., 3′ UTR SNPs) on miRNA target site recognition. Our study sought evidence for the role of genetic variation in modulating miRNA expression variation.

Importantly, our findings can be integrated into studies of complex phenotypes from genome-wide association studies and sequencing efforts. We investigated published associations between genetic variation and disease susceptibility or quantitative traits from the NHGRI repository. For example, we identified two SNPs, rs4598195 and rs4730276, that have been found to be associated with ulcerative colitis [41,42] and that predict the expression of hsa-miR-629*; remarkably, hsa-miR-629 has been independently found to be differentially expressed in ulcerative colitis [43]. Two independent SNPs, rs7191888 on chromosome 16 and rs10259085 on chromosome 7, are reported associations with multiple sclerosis [44] and associated with the expression of hsa-miR-126* and hsa-miR-126 respectively in our dataset. A SNP rs6085920 has been associated with serum uric acid [45] and is associated with the expression of miRNA hsa-miR-141, which has been proposed as a therapeutic target for the prevention of progressive kidney disease [46].

In a similar vein, we systematically investigated published clinical pharmacogenetic associations for their role in regulating miRNA expression. A SNP rs4888024 was found to be associated with end-of-induction minimal residual disease in childhood acute lymphoblastic leukemia from 2 independent cohorts and higher methotrexate clearance [47]. Leukemias with rearrangement of the *MLL* gene have been shown to be characterized by the absence of hsa-miR-340 expression [48]. Our miR-eQTL data support a relationship (p = 8.5x10^-4^) between rs4888024 and the expression of hsa-miR-340. Furthermore, a SNP rs730012 (in *LTC4S*) has been found to be associated with exacerbation rates in asthma patients treated with montelukast [49]. Our data support a relationship between this SNP and the expression of miR-146b (p = 3.89x10^-4^), which has been implicated in asthma pathogenesis in murine models of acute and chronic asthma [50]. As the detailed mechanisms underlying numerous genotype-phenotype correlations in both disease genomics and pharmacogenomics remain largely unknown, our study provides important hypotheses for future investigations. To this end, we make the results of our study available to the scientific community through an online public resource [51].

Using Q-PCR, we were able to confirm the expression of two miRNAs and two mRNAs, the inverse correlations between these miRNAs and mRNAs, and an identified miR-eQTL, despite the limited sample size due to the availability of RNA. Although the biological interactions between the miRNAs and the mRNAs will require extensive experimental validation (e.g. cloning, transfection etc.), our study generates, in aggregate, numerous hypotheses that warrant continued investigations and that may have substantial impact on the study of human diseases as well as on pharmacogenetics.

## Conclusion

Our comprehensive catalog of miR-eQTLs in liver suggests numerous functional links to important disease traits and drug response phenotypes. The discovery of genetic variations that influence miRNA expression (and thus the expression of mRNA targets) facilitates a genomic annotation approach that is likely to lead to more robust associations between variants and complex human phenotypes.

## Methods

### mRNA expression analysis

Gene expression profiling in liver was done on 206 samples. The liver samples were mostly derived from donor livers not used for whole organ transplants. The study described here was made possible by liver samples from deceased anonymous individuals; thus, for the purpose of this study, the utilized livers did not involve “human subjects.” Genotyping on these samples was performed on the Illumina Human 610 quad beadchip platform (GPL8887) with Bimbam, as previously described [17]. Array hybridizations using the Agilent 4x44 arrays were conducted at The University of Chicago according to manufacturer’s instructions. The quantification of signals, the normalization approach used, and other quality control procedures performed as well as the subsequent mRNA-level analyses, including the covariate modeling, surrogate variable analysis [52], and eQTL mapping, were previously described [17]. The mRNA data have been deposited into Gene Expression Omnibus (GSE28893).

### Samples

MiRNA expression was measured in 79 of the liver samples using the Exiqon miRCURY™ LNA Array v10.0 (for approximately 850 miRNAs) (Exiqon, Inc., Denmark). These 79 samples were a subset of the 206 liver tissue samples used for the mRNA expression profiling. The collection of samples from the Liver Tissue Cell Distribution System (funded by NIH #N01-DK-7-0004/HHSN267200700004C and by the Cooperative Human Tissue Network) was approved by the institutional review boards (IRBs); The University of Chicago IRB also approved the use of the samples for the study described here.

### miRNA expression profiling

Total RNA was extracted using TRIzol reagent according to manufacturer’s instructions (Invitrogen, Carlsbad, CA), followed by RNeasy Mini Kit cleanup (Qiagen, Valencia, CA). Cleanup protocol was modified to preserve microRNA (modification instructions provided by Exiqon). Sample RNA quality control was performed using Bioanalyzer2100. In addition to the rRNA ratio (s28/s18), the bioanalyzer evaluates the quality of the RNA using RNA Integrity Number (RIN); RIN > 7 was used as threshold. Array hybridizations were performed by Exiqon. Quantified signals were background corrected through *normexp* with offset value 10 based on a convolution model [53]. Normalization of quantified signals was done using the global Lowess (LOcally WEighted Scatterplot Smoothing) regression algorithm [54]. Quantified miRNA expression levels were log_2_-transformed.

### miRNA-mRNA associations

Linear regression analyses were performed between the log_2_-transformed miRNA (n = 277) expression and the quantile-normalized mRNA (n = 19,749) expression. The distribution of p-values for those comparisons with a negative correlation coefficient was plotted, showing an enrichment towards low p-values for the miRNA-mRNA correlations. For multiple testing adjustment, an FDR approach was used [19].

### miRNA eQTL mapping

We conducted genome-wide association studies to map miRNA expression to genomic loci as miRNA eQTLs (miR-eQTLs). We had performed imputation on the 206 samples (and thus on the subset [n = 79] of miRNA samples) to increase the number of interrogated SNPs, as previously described [17]. Each miRNA expression phenotype, considered as a quantitative trait, was tested for association with genome-wide markers (n = 1,707,239) using linear regression. In the covariate modeling, age, sex, and the first 3 (genotype-based) principal components were used as covariates if they were associated with the miRNA expression trait. In the QTL mapping, we filtered for SNPs that failed to meet the minor allele frequency threshold of >15% and showed significant deviation from Hardy-Weinberg equilibrium (Fisher’s exact test, p < 0.001). To ensure the robustness of our findings to the presence of unknown hidden factors, we utilized the probabilistic estimation of expression residuals (PEER) framework [55], which infers hidden determinants of expression levels and generates a residual expression profile. From the diagnostic plot of the factor relevance [55], we used 4 inferred factors and performed an eQTL scan on the residual dataset. For the miRNAs considered in this study, the median correlation between the pre- and post- hidden factor adjusted miRNA levels is 0.86 (with minimum correlation of 0.70 and maximum of 0.996).

### Enrichment analyses

To test for enrichment of mRNA eQTLs (or pharmacogenetic associations) among the miR-eQTLs, we conducted simulations as previously described [4]. Briefly, we generated 1000 sets of SNPs matching the allele frequency distribution of the miRNA-associated SNPs. For each such set, we determined the number of mRNA-associated SNPs (at a given p-value threshold). The overlap of each set with the list of mRNA-associated SNPs yields an empirical null distribution, allowing us to determine the expected overlap count. The proportion of the simulated sets with overlap count that matches or exceeds the actual observed overlap between the miRNA-associated SNPs and the mRNA-associated SNPs provides an empirical p-value for the enrichment.

### Q-PCR Confirmation

To confirm the expression of select miRNAs and mRNAs, the correlations between the miRNAs and mRNAs, and the miR-eQTLs, quantitative PCR (Q-PCR) studies of two miRNAs (miR-148a and miR-185a) and two mRNAs (*PTGIS* and *ADRB2*) were conducted. We performed correlation analyses between the Q-PCR and microarray data, between the miRNAs and mRNAs, and between the miR-eQTLs and the miRNAs. The Q-PCR confirmation was performed in the samples for which total RNA was still available (n = 53). Q-PCR for miRNAs was performed with Taqman MicroRNA Assays (Invitrogen, CA, USA) using ViiA™ 7 Real-Time PCR System (Invitrogen) according to the manufacturer’s instructions. The *U6* gene (*RNU6B*) was used as an internal control. Q-PCR for the two mRNA genes was conducted using iQ™ SYBR® Green Supermix (Bio-Rad, CA, USA) according to the protocol developed in our previously study [56]. The ribosomal *18S* RNA gene was used as an internal control for the normalization of the mRNA expression. Primer sequences for *PTGIS* and *ADRB2* genes are: PTGIS_F: 5′-CAGCTCCAAGTCCAAGTGCA-3′, PTGIS_R: 5′-CACTGCCTGGGGAGGAGTTAT-3′; and ADRB2_F: 5′-GGACTTCCATTGATGTGCTGT-3′, ADRB2_R: 5′-GTCAGCAGGCTCTGGTACTTG-3′, respectively. Annealing temperature used for the Q-PCR reactions for both genes was 65°C. The relative expression levels between the quantified miRNA or mRNA genes and the respective internal control genes were used in the data analyses.

Correlation analyses were conducted using the SPSS 20.0 program (SPSS Inc., IL, USA), and data were plotted using Graphpad Prism 6.0 (Graphpad Software, CA, USA). P < 0.05 was used as a cut-off for statistical significance.

## Abbreviations

FDR: False discovery rate; HIV: Human immunodeficiency virus; IRB: Institutional review board; Lowess: Locally Weighted Scatterplot Smoothing; miR-eQTLs: microRNA expression quantitative trait loci; NHGRI: National Human Genome Research Institute; PACdb: Pharmacogenetics-Cell line database; PEER: Probabilistic estimation of expression residuals; PharmGKB: The Pharmacogenomics Knowledge Base; PK: Pharmacokinetic(s); QTL: Quantitative trait loci; RIN: RNA integrity number; SNP: Single nucleotide polymorphism; UTR: Untranslated region; VIP: Very Important Pharmacogenes.

## Competing interests

The authors declare that they have no competing interests.

## Authors’ contributions

ERG carried out the data analysis, participated in the data interpretation, and drafted the manuscript. FI participated in the design of the study and provided the genotyping and transcriptome data. RW conducted Q-PCR for the validation experiments. LW carried out data analysis for the validation study. ZM participated in the design of the validation study. SM carried out the RNA sample extraction and quality control. JR participated in the sample preparation. RSH participated in the design of the data analysis plan. NJC participated in the design of the data analysis plan and conceived of the data interpretation. MJR participated in the design and coordination of the study, and helped draft the manuscript. WL conceived of the study, originally designed and coordinated the study, and helped draft the manuscript. All authors read and approved the final manuscript.

## Supplementary Material

Additional file 1: Figure S1 A heatmap illustrating a two-way hierarchical clustering of miRNAs and samples. Click here for file

Additional file 2: Table S1 Top miRNA-mRNA associations in liver. Click here for file

Additional file 3: Table S2 The most significant miRNA-mRNA associations and overlap with computational miRNA prediction approaches. Click here for file

Additional file 4: Figure S2 The miRNA-associated SNPs are more likely to be mRNA-associated than a random set of allele frequency matched SNPs. Click here for file

Additional file 5: Table S3 NHGRI Catalog SNPs and miRNA associations. Click here for file

Additional file 6: Table S4 Significant associations between miRNAs and VIP genes. Click here for file

Additional file 7: Figure S3 A comparison of miRNA effect on VIP genes and the global distribution of effect sizes for the negative associations (FDR<0.05) between miRNAs and mRNAs. Click here for file

Additional file 8: Figure S4 A comparison of the distribution of effect sizes on target mRNAs for the miRNAs associated (p < 0.001) with the pharmacogenetic variants and the remaining expressed miRNAs. Click here for file

## References

[B1] Yu YP Gene expression alterations in prostate cancer predicting tumor aggression and preceding development of malignancy J Clin Oncol 2004 22 14 2790 2799 15254046 10.1200/JCO.2004.05.158

[B2] Xu B Karayiorgou M Gogos JA MicroRNAs in psychiatric and neurodevelopmental disorders Brain Res 2010 1338 78 88 20388499 10.1016/j.brainres.2010.03.109 PMC2883644

[B3] Pauley KM Cha S Chan EK MicroRNA in autoimmunity and autoimmune diseases J Autoimmun 2009 32 3–4 189 194 19303254 10.1016/j.jaut.2009.02.012 PMC2717629

[B4] Nicolae DL Trait-associated SNPs are more likely to be eQTLs: annotation to enhance discovery from GWAS PLoS Genet 2010 6 4 e1000888 20369019 10.1371/journal.pgen.1000888 PMC2848547

[B5] Ambros V MicroRNAs and other tiny endogenous RNAs in C. elegans Curr Biol 2003 13 10 807 818 12747828 10.1016/s0960-9822(03)00287-2

[B6] Brennecke J bantam encodes a developmentally regulated microRNA that controls cell proliferation and regulates the proapoptotic gene hid in Drosophila Cell 2003 113 1 25 36 12679032 10.1016/s0092-8674(03)00231-9

[B7] Lee RC Feinbaum RL Ambros V The C. elegans heterochronic gene lin-4 encodes small RNAs with antisense complementarity to lin-14 Cell 1993 75 5 843 854 8252621 10.1016/0092-8674(93)90529-y

[B8] Harfe BD The RNaseIII enzyme Dicer is required for morphogenesis but not patterning of the vertebrate limb Proc Natl Acad Sci USA 2005 102 31 10898 10903 16040801 10.1073/pnas.0504834102 PMC1182454

[B9] Taganov KD NF-kappaB-dependent induction of microRNA miR-146, an inhibitor targeted to signaling proteins of innate immune responses Proc Natl Acad Sci USA 2006 103 33 12481 12486 16885212 10.1073/pnas.0605298103 PMC1567904

[B10] Chen XM MicroRNA signatures in liver diseases World J Gastroenterol 2009 15 14 1665 1672 19360909 10.3748/wjg.15.1665 PMC2668771

[B11] Murakami Y Comprehensive analysis of microRNA expression patterns in hepatocellular carcinoma and non-tumorous tissues Oncogene 2006 25 17 2537 2545 16331254 10.1038/sj.onc.1209283

[B12] Chu AS Friedman JR A role for microRNA in cystic liver and kidney diseases J Clin Invest 2008 118 11 3585 3587 18949060 10.1172/JCI36870 PMC2571036

[B13] Krutzfeldt J Silencing of microRNAs in vivo with ‘antagomirs’ Nature 2005 438 7068 685 689 16258535 10.1038/nature04303

[B14] Klein TE Integrating genotype and phenotype information: an overview of the PharmGKB project, Pharmacogenetics Research Network and Knowledge Base Pharmacogenomics J 2001 1 3 167 170 11908751 10.1038/sj.tpj.6500035

[B15] Chang J miR-122, a mammalian liver-specific microRNA, is processed from hcr mRNA and may downregulate the high affinity cationic amino acid transporter CAT-1 RNA Biol 2004 1 2 106 113 17179747 10.4161/rna.1.2.1066

[B16] Eisen MB Cluster analysis and display of genome-wide expression patterns Proc Natl Acad Sci USA 1998 95 25 14863 14868 9843981 10.1073/pnas.95.25.14863 PMC24541

[B17] Innocenti F Identification, replication, and functional fine-mapping of expression quantitative trait loci in primary human liver tissue PLoS Genet 2011 7 5 e1002078 21637794 10.1371/journal.pgen.1002078 PMC3102751

[B18] Doench JG Sharp PA Specificity of microRNA target selection in translational repression Genes Dev 2004 18 5 504 511 15014042 10.1101/gad.1184404 PMC374233

[B19] Storey JD Tibshirani R Statistical significance for genomewide studies Proc Natl Acad Sci USA 2003 100 16 9440 9445 12883005 10.1073/pnas.1530509100 PMC170937

[B20] Griffiths-Jones S miRBase: microRNA sequences, targets and gene nomenclature Nucleic Acids Res 2006 34 Database issue) D140-4 16381832 10.1093/nar/gkj112 PMC1347474

[B21] Gamazon ER Exprtarget: an integrative approach to predicting human microRNA targets PLoS One 2010 5 10 e13534 20975837 10.1371/journal.pone.0013534 PMC2958831

[B22] Huang Da W Sherman BT Lempicki RA Bioinformatics enrichment tools: paths toward the comprehensive functional analysis of large gene lists Nucleic Acids Res 2009 37 1 1 13 19033363 10.1093/nar/gkn923 PMC2615629

[B23] Benjamini Y Controlling the false discovery rate in behavior genetics research Behav Brain Res 2001 125 1–2 279 284 11682119 10.1016/s0166-4328(01)00297-2

[B24] The Gene Ontology’s Reference Genome Project a unified framework for functional annotation across species PLoS Comput Biol 2009 5 7 e1000431 19578431 10.1371/journal.pcbi.1000431 PMC2699109

[B25] Scheet P Stephens M A fast and flexible statistical model for large-scale population genotype data: applications to inferring missing genotypes and haplotypic phase Am J Hum Genet 2006 78 4 629 644 16532393 10.1086/502802 PMC1424677

[B26] Pruim RJ LocusZoom: regional visualization of genome-wide association scan results Bioinformatics 2010 26 18 2336 2337 20634204 10.1093/bioinformatics/btq419 PMC2935401

[B27] Rosenfeld N MicroRNAs accurately identify cancer tissue origin Nat Biotechnol 2008 26 4 462 469 18362881 10.1038/nbt1392

[B28] McDonagh EM From pharmacogenomic knowledge acquisition to clinical applications: the PharmGKB as a clinical pharmacogenomic biomarker resource Biomark Med 2011 5 6 795 806 22103613 10.2217/bmm.11.94 PMC3339046

[B29] Gamazon ER PACdb: a database for cell-based pharmacogenomics Pharmacogenet Genomics 2010 20 4 269 273 20216476 10.1097/FPC.0b013e328337b8d6 PMC2914089

[B30] Ross CJ Genetic variants in TPMT and COMT are associated with hearing loss in children receiving cisplatin chemotherapy Nat Genet 2009 41 12 1345 1349 19898482 10.1038/ng.478

[B31] Glynn SA A mitochondrial target sequence polymorphism in manganese superoxide dismutase predicts inferior survival in breast cancer patients treated with cyclophosphamide Clin Cancer Res 2009 15 12 4165 4173 19509150 10.1158/1078-0432.CCR-09-0119 PMC2697269

[B32] Huang RS Population differences in microRNA expression and biological implications RNA Biol 2011 8 4 692 701 21691150 10.4161/rna.8.4.16029 PMC3225983

[B33] Gamazon ER Badner JA Cheng L Zhang C Zhang D Cox NJ Gershon ES Kelsoe JR Greenwood TA Nievergelt CM Chen C McKinney R Shilling PD Schork NJ Smith EN Bloss CS Nurnberger JI Edenberg HJ Foroud T Koller DL Scheftner WA Coryell W Rice J Lawson WB Nwulia EA Hipolito M Byerley W McMahon FJ Schulze TG Berrettini WH Enrichment of cis-regulatory gene expression SNPs and methylation quantitative trait loci among bipolar disorder susceptibility variants Mol Psychiatry 2013 18 3 340 346 22212596 10.1038/mp.2011.174 PMC3601550

[B34] Below JE Genome-wide association and meta-analysis in populations from Starr County, Texas, and Mexico City identify type 2 diabetes susceptibility loci and enrichment for expression quantitative trait loci in top signals Diabetologia 2011 54 8 2047 2055 21647700 10.1007/s00125-011-2188-3 PMC3761075

[B35] Rogler CE MicroRNA-23b cluster microRNAs regulate transforming growth factor-beta/bone morphogenetic protein signaling and liver stem cell differentiation by targeting Smads Hepatology 2009 50 2 575 584 19582816 10.1002/hep.22982

[B36] Parsons JT Horwitz AR Schwartz MA Cell adhesion: integrating cytoskeletal dynamics and cellular tension Nat Rev Mol Cell Biol 2010 11 9 633 643 20729930 10.1038/nrm2957 PMC2992881

[B37] Valastyan S Weinberg RA Roles for microRNAs in the regulation of cell adhesion molecules J Cell Sci 2011 124 Pt 7 999 1006 21402873 10.1242/jcs.081513 PMC3056602

[B38] van Rooij E Dysregulation of microRNAs after myocardial infarction reveals a role of miR-29 in cardiac fibrosis Proc Natl Acad Sci USA 2008 105 35 13027 13032 18723672 10.1073/pnas.0805038105 PMC2529064

[B39] Ventura A Jacks T MicroRNAs and cancer: short RNAs go a long way Cell 2009 136 4 586 591 19239879 10.1016/j.cell.2009.02.005 PMC3910108

[B40] Valastyan S A pleiotropically acting microRNA, miR-31, inhibits breast cancer metastasis Cell 2009 137 6 1032 1046 19524507 10.1016/j.cell.2009.03.047 PMC2766609

[B41] Silverberg MS Ulcerative colitis-risk loci on chromosomes 1p36 and 12q15 found by genome-wide association study Nat Genet 2009 41 2 216 220 19122664 10.1038/ng.275 PMC2652837

[B42] McGovern DP Genome-wide association identifies multiple ulcerative colitis susceptibility loci Nat Genet 2010 42 4 332 337 20228799 10.1038/ng.549 PMC3087600

[B43] Wu F MicroRNAs are differentially expressed in ulcerative colitis and alter expression of macrophage inflammatory peptide-2 alpha Gastroenterology 2008 135 5 1624 1635 e24 18835392 10.1053/j.gastro.2008.07.068

[B44] Baranzini SE Genome-wide association analysis of susceptibility and clinical phenotype in multiple sclerosis Hum Mol Genet 2009 18 4 767 778 19010793 10.1093/hmg/ddn388 PMC4334814

[B45] McArdle PF Association of a common nonsynonymous variant in GLUT9 with serum uric acid levels in old order amish Arthritis Rheum 2008 58 9 2874 2881 18759275 10.1002/art.23752 PMC2779583

[B46] Wang B miR-200a Prevents renal fibrogenesis through repression of TGF-beta2 expression Diabetes 2011 60 1 280 287 20952520 10.2337/db10-0892 PMC3012183

[B47] Yang JJ Genome-wide interrogation of germline genetic variation associated with treatment response in childhood acute lymphoblastic leukemia JAMA 2009 301 4 393 403 19176441 10.1001/jama.2009.7 PMC2664534

[B48] Dixon-McIver A Distinctive patterns of microRNA expression associated with karyotype in acute myeloid leukaemia PLoS One 2008 3 5 e2141 18478077 10.1371/journal.pone.0002141 PMC2373886

[B49] Lima JJ Influence of leukotriene pathway polymorphisms on response to montelukast in asthma Am J Respir Crit Care Med 2006 173 4 379 385 16293801 10.1164/rccm.200509-1412OC PMC2662939

[B50] Garbacki N MicroRNAs profiling in murine models of acute and chronic asthma: a relationship with mRNAs targets PLoS One 2011 6 1 e16509 21305051 10.1371/journal.pone.0016509 PMC3030602

[B51] Gamazon ER SCAN: SNP and copy number annotation Bioinformatics 2010 26 2 259 262 19933162 10.1093/bioinformatics/btp644 PMC2852202

[B52] Leek JT Storey JD Capturing heterogeneity in gene expression studies by surrogate variable analysis PLoS Genet 2007 3 9 1724 1735 17907809 10.1371/journal.pgen.0030161 PMC1994707

[B53] Ritchie ME A comparison of background correction methods for two-colour microarrays Bioinformatics 2007 23 20 2700 2707 17720982 10.1093/bioinformatics/btm412

[B54] Cleveland WS Robust locally weighted regression and smoothing scatterplots J Amer Statist Assoc 1979 74 829 836

[B55] Stegle O Using probabilistic estimation of expression residuals (PEER) to obtain increased power and interpretability of gene expression analyses Nat Protoc 2012 7 3 500 507 22343431 10.1038/nprot.2011.457 PMC3398141

[B56] Liu W Interactions between MDM2 and TP53 Genetic alterations, and their impact on response to MDM2 inhibitors and other chemotherapeutic drugs in cancer cells Clin Cancer Res 2009 15 24 7602 7607 19996219 10.1158/1078-0432.CCR-09-0890 PMC2794936

