# Supplementary figures and images for: A genome-wide integrative study of microRNAs in human liver
Source: BMC Genomics. 2013 Jun 13;14:395. doi: 10.1186/1471-2164-14-395 (PMC3710218; doi:10.1186/1471-2164-14-395)

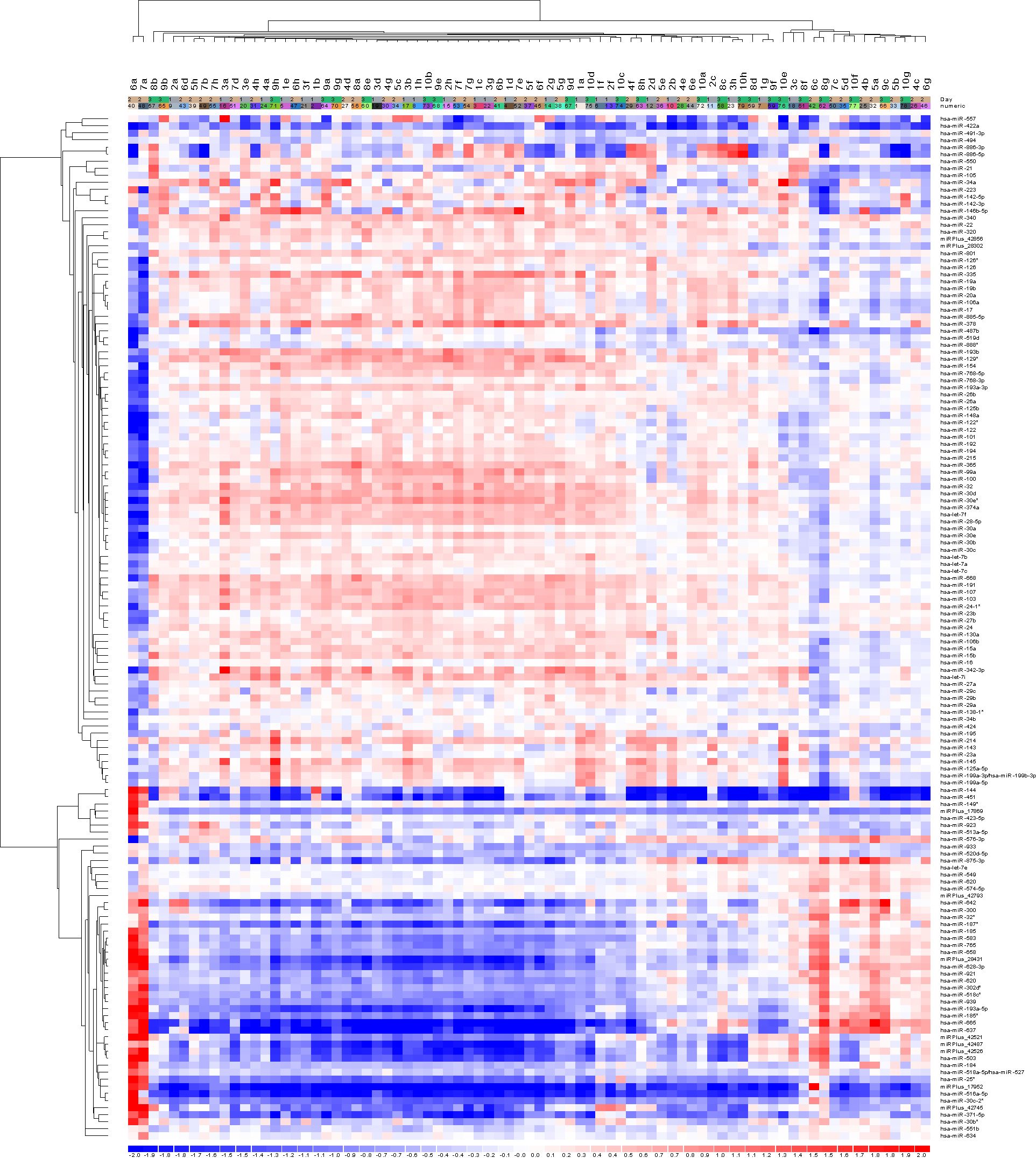

Supplement: Additional file 1: Figure S1 — A heatmap illustrating a two-way hierarchical clustering of miRNAs and samples. [file 1471-2164-14-395-S1.jpeg]

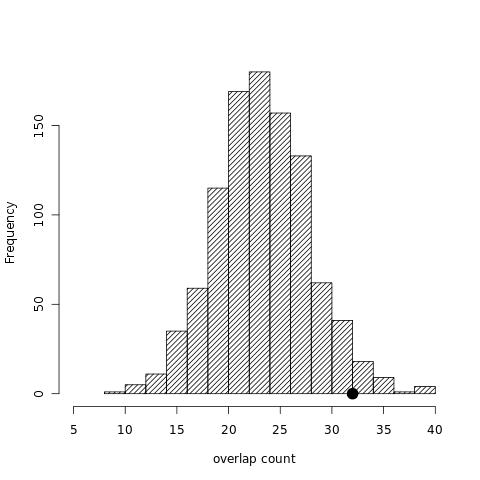

Supplement: Additional file 4: Figure S2 — The miRNA-associated SNPs are more likely to be mRNA-associated than a random set of allele frequency matched SNPs. [file 1471-2164-14-395-S4.jpeg]

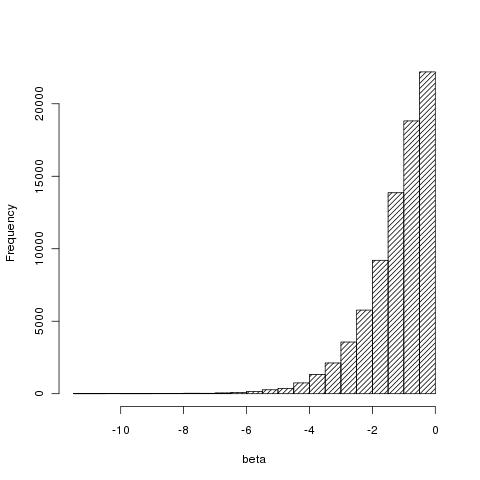

Supplement: Additional file 7: Figure S3 — A comparison of miRNA effect on VIP genes and the global distribution of effect sizes for the negative associations (FDR<0.05) between miRNAs and mRNAs. [file 1471-2164-14-395-S7.jpeg]

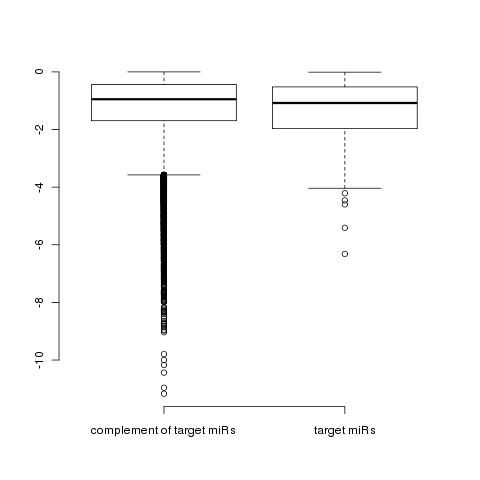

Supplement: Additional file 8: Figure S4 — A comparison of the distribution of effect sizes on target mRNAs for the miRNAs associated (p < 0.001) with the pharmacogenetic variants and the remaining expressed miRNAs. [file 1471-2164-14-395-S8.jpg]
